# Vildagliptin-induced acute lung injury: a case report

**DOI:** 10.1186/s13256-016-1006-4

**Published:** 2016-08-12

**Authors:** Nobumasa Ohara, Masanori Kaneko, Kazuhiro Sato, Ryoko Maruyama, Tomoyasu Furukawa, Junta Tanaka, Kenzo Kaneko, Kyuzi Kamoi

**Affiliations:** 1 Department of Endocrinology and Metabolism, Nagaoka Red Cross Hospital, 2-297-1 Senshu, Nagaoka, Niigata 940-2085 Japan; 2 Department of Endocrinology and Metabolism, Uonuma Institute of Community Medicine, Niigata University Medical and Dental Hospital, Niigata, Japan; 3 Department of Respiratory Medicine, Nagaoka Red Cross Hospital, Niigata, Japan; 4 Department of Pharmaceuticals, Nagaoka Red Cross Hospital, Niigata, Japan; 5 Department of Pharmaceuticals, Niitsu Medical Center Hospital, Niigata, Japan; 6 Department of Respiratory Medicine and Infectious Disease, Niigata University Medical and Dental Hospital, Niigata, Japan; 7 Center of Diabetes, Endocrinology and Metabolism, Joetsu General Hospital, Niigata, Japan

**Keywords:** Dipeptidyl peptidase-4 inhibitor, Acute respiratory failure, Ground-glass opacity, Elevated pancreatic enzyme, Diabetes mellitus, Human leukocyte antigen, Leukocyte migration test

## Abstract

**Background:**

Dipeptidyl peptidase-4 inhibitors are a class of oral hypoglycemic drugs and are used widely to treat type 2 diabetes mellitus in many countries. Adverse effects include nasopharyngitis, headache, elevated serum pancreatic enzymes, and gastrointestinal symptoms. In addition, a few cases of interstitial pneumonia associated with their use have been reported in the Japanese literature. Here we describe a patient who developed drug-induced acute lung injury shortly after the administration of the dipeptidyl peptidase-4 inhibitor vildagliptin.

**Case presentation:**

A 38-year-old Japanese woman with diabetes mellitus developed acute respiratory failure 1 day after administration of vildagliptin. Chest computed tomography revealed nonsegmental ground-glass opacities in her lungs. There was no evidence of bacterial pneumonia or any other cause of her respiratory manifestations. After discontinuation of vildagliptin, she recovered fully from her respiratory disorder. She received insulin therapy for her diabetes mellitus, and her subsequent clinical course has been uneventful.

**Conclusions:**

The period of drug exposure in previously reported cases of patients with drug-induced interstitial pneumonia caused by dipeptidyl peptidase-4 inhibitor varied from several days to over 6 months. In the present case, our patient developed interstitial pneumonia only 1 day after the administration of vildagliptin. The precise mechanism of her vildagliptin-induced lung injury remains uncertain, but physicians should consider that dipeptidyl peptidase-4 inhibitor-induced lung injury, although rare, may appear acutely, even within days after administration of this drug.

## Background

Dipeptidyl peptidase-4 (DPP-4) inhibitors, also called gliptins, are a relatively new class of oral hypoglycemic drugs with proven efficacy, tolerability, and safety [1, 2]. They are used widely to treat type 2 diabetes mellitus in many countries. Adverse effects include nasopharyngitis, headache, elevated serum pancreatic enzymes, gastrointestinal symptoms, and infrequent urticaria, angioedema, and hypersensitivity reactions. Overall, there have been few reports on drug-induced lung injury caused by DPP-4 inhibitors. However, several cases of interstitial pneumonia (IP) associated with the use of DPP-4 inhibitors have been reported in the medical literature in Japanese [3–5].

Here we report a case of acute lung injury that occurred shortly after the administration of a DPP-4 inhibitor, vildagliptin.

## Case presentation

A 38-year-old Japanese woman was admitted to our hospital because of coma after several hours of dyspnea and hyperpnea. Her medical and family histories were unremarkable. She had smoked 10 cigarettes per day for 18 years (9 pack years of tobacco smoking). She developed thirst and polyuria 2 months before admission. The day before admission, she visited a local clinic because of persistent thirst, polyuria, and a 10-kg body weight loss over the past 2 months. Her height, body weight, and body temperature were 158 cm, 51 kg, and 36.8 °C, respectively. Blood chemistry showed a high fasting plasma glucose level of 15.2 mmol/L, glycated hemoglobin (HbA1c) of 9.8 %, and a normal white blood cell (WBC) count of 5600/μL. A urine analysis was not performed. She was diagnosed as having diabetes mellitus and prescribed a DPP-4 inhibitor, vildagliptin, at 100 mg/day (50 mg in the morning and 50 mg in the evening), which she started taking that evening. In the midmorning of the day of admission, she complained of nausea and vomiting; by the evening, she had developed dyspnea and hyperpnea without sputum.

**Fig. 1:**
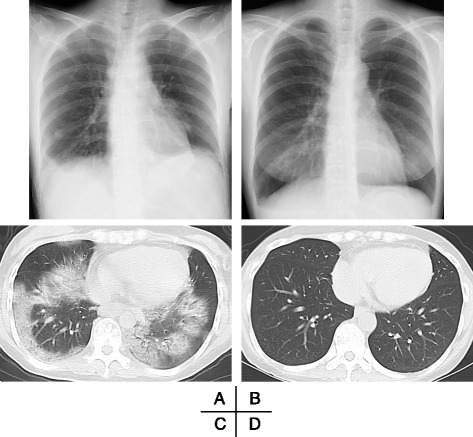
Radiologic findings. A chest X-ray performed on admission with the patient in the supine position showed a nodule-like shadow in the lower lobe of her right lung and reticular shadows in the lower lobes of both lungs (**a**). A chest X-ray performed on day 10 after admission with the patient in the sitting position showed improvements in the right nodular shadow and reticular shadows of the lower lobes of both lungs (**b**). Chest computed tomography performed on admission showed subpleural-predominant, nonsegmental ground-glass opacities in the lower lobes of both lungs (**c**). Chest computed tomography performed on day 19 after admission showed improvements in the ground-glass opacities in both lungs (**d**)

On admission, she was comatose and breathing deeply (24 breaths/minute). Her body weight, body temperature, blood pressure, and heart rate were 49 kg, 35.7 °C, 91/54 mmHg, and 123 beats/minute, respectively. Her oral cavity and skin were dry. No eruption, wheezing, chest rales, or heart murmurs were detected; however, the breath sounds in the lower lobes of her lungs were decreased. Arterial blood gas analysis showed severe metabolic acidosis and a decreased partial oxygen pressure with impaired pulmonary diffusion (Table 1). Blood chemistry revealed a high WBC count, hyperglycemia, and ketonemia. Serum creatinine, potassium, lactate dehydrogenase (LDH), and C-reactive protein (CRP) were high. In addition, her serum levels of pancreatic enzymes, such as amylase, lipase, trypsin, and phospholipase A2, were high. A chest X-ray showed abnormal shadows in the lower lobes of her lungs (Fig. 1a). Computed tomography (CT) showed ground-glass opacities in the lower lobes of her lungs (Fig. 1c); no abnormality in her liver, pancreas, or kidneys was detected. Blood culture and urinary antigens of *Streptococcus pneumoniae* and *Legionella pneumophila* were both negative. Her serum β-D-glucan levels were normal. She tested negative for anti-*Mycoplasma pneumoniae* antibody, anti-*Trichosporon asahii* antibody, anti-nuclear antibody, and rheumatoid factor.

**Table 1:** Laboratory findings at the time of admission in July 2013

Hematology		
Red blood cells	510×10^4^/μL	(427–571)
Hemoglobin	15.2 g/dL	(12.4–17.2)
Hematocrit	45.3 %	(38.7–50.3)
White blood cells	28,840/μL	(4000–9000)
Neutrophils	89.2 %	(36.0–71.0)
Eosinophils	0.0 %	(<11.0)
Basophils	0.2 %	(<1.5)
Monocytes	6.8 %	(<10.0)
Lymphocytes	3.9 %	(20.0–50.0)
Platelets	30.9×10^4^/μL	(12.0–30.0)
Chemistry		
Casual plasma glucose	49.8 mmol/L	(3.9–7.8)
HbA1c	10.2 %	(4.6–6.2)
Acetoacetate	1970 μmol/L	(<55)
3-Hydroxybutyrate	5335 μmol/L	(<85)
Total protein	7.0 g/dL	(6.7–8.3)
Albumin	4.3 g/dL	(3.8–5.3)
Aspartate aminotransferase	17 IU/L	(13–33)
Alanine aminotransferase	19 IU/L	(6–27)
Lactate dehydrogenase	262 IU/L	(105–215)
Alkaline phosphatase	336 IU/L	(115–359)
Amylase	124 IU/L	(41–112)
Lipase	138 U/L	(5–35)
Trypsin	995 ng/mL	(100–550)
Phospholipase A2	5160 ng/dL	(130–400)
Elastase-1	142 ng/dL	(0–300)
Urea nitrogen	25.9 mg/dL	(8.0–22.0)
Creatinine	1.57 mg/dL	(0.4–0.7)
Sodium	125 mmol/L	(137–147)
Potassium	7.1 mmol/L	(3.5–4.7)
Chloride	98 mmol/L	(98–108)
C-reactive protein	2.14 mg/dL	(<0.30)
KL-6	195 U/mL	(<490)
Arterial blood gas analysis under room air		
pH	6.886	(7.35–7.45)
Partial carbon dioxide pressure	9.0 mmHg	(32–48)
Partial oxygen pressure	83.1 mmHg	(83–108)
Bicarbonate	1.6 mmol/L	(21–28)
Saturation of arterial oxygen	94.3 %	(95–99)
Alveolar-arterial oxygen difference	55.9 mmHg	(5–10)

The reference range for each parameter is shown in parentheses.

*HbA1c* glycated hemoglobin, *KL-6* sialylated carbohydrate antigen Krebs von den Lungen-6

She was diagnosed as having diabetic ketoacidosis (DKA) and acute renal failure and received intravenous saline and insulin. Her pulmonary problems were initially treated with a course of empiric antibiotic therapy with 0.75 g/day of intravenous meropenem and oxygen inhalation.

The next day, she regained consciousness and was no longer dyspneic. Her body temperature (36.8 °C), blood pressure (112/74 mmHg), respiration rate (18 breaths/minute), arterial pH (7.37), and partial oxygen pressure (86.3 mmHg) had normalized without the need for oxygen inhalation. Her plasma glucose had fallen to 13.9 mmol/L, and her electrolytes were normal.

She began subcutaneous insulin injection therapy for her diabetes mellitus on day 3 and did not resume taking vildagliptin. Blood chemistry findings on day 5 were: a WBC count of 4050/μL, serum CRP 0.33 mg/dL, LDH 222 IU/L, and creatinine 0.68 mg/dL. Because her respiratory problems resolved, the meropenem was discontinued that day. Her serum CRP (0.09 mg/dL) and LDH (177 IU/L) levels normalized on day 10. A chest X-ray showed improvements in the reticular shadows (Fig. 1b). Chest CT performed on day 19 found fewer ground-glass opacities (Fig. 1d). A paired serum antiviral antibody test detected no elevation in antibody titers to respiratory syncytial virus, parainfluenza virus 1 to 3, influenza virus A and B, adenovirus, Epstein–Barr virus, human herpes virus, cytomegalovirus, or herpes simplex virus.

Her serum C-peptide levels were low (<0.2 ng/mL) before and after intravenous glucagon load on day 7 of admission. She tested negative for glutamic acid decarboxylase antibody (<1.5 U/mL) but positive for insulinoma-associated antigen 2 autoantibody (3.1 U/mL; reference range, <0.4 U/mL). These findings indicated a diagnosis of type 1 diabetes mellitus (T1D). Human leukocyte antigen (HLA) typing showed A*11:01/31:01, B*39:02/54:01, and C*01:02/07:02 class I genes and DRB1*04:05/13:02, DQB1*04:01/06:04, DQA1*01:02/03:03, and DPB1*04:01/05:01 class II genes.

Serum levels of amylase (234 IU/L), lipase (201 U/L), trypsin (2460 ng/mL), phospholipase A2 (1270 ng/dL), and elastase-1 (1252 ng/dL) remained high on day 10, but she had no abdominal pain and ultrasonography detected no abnormality in her pancreas. All of these pancreatic enzymes (42 IU/L, 24 U/L, 299 ng/mL, 330 ng/dL, and 142 ng/dL, respectively) normalized on day 28 of admission.

To examine the possible association of vildagliptin administration with her elevated pancreatic enzymes and acute lung injury, a cell-mediated immunity assessment was performed. Vildagliptin yielded negative results in a drug-induced lymphocyte stimulation test (DLST; stimulation index 1.1, reference range <1.6) but positive results in a leukocyte migration test (LMT; migration index 1.6, reference range 0.6 to 1.4).

After participating in a self-management diabetes mellitus education program, she was discharged on day 32. She continued insulin therapy in the out-patient clinic of our hospital, and her subsequent clinical course has been uneventful.

## Discussion

A Japanese woman with newly diagnosed diabetes mellitus began treatment with vildagliptin. The next day, she was admitted to our hospital because of acute respiratory failure with abnormal lung shadows in a diabetic coma. She regained her consciousness after correction of DKA. Because her diabetes mellitus turned out to be T1D, she received subcutaneous insulin injection therapy and did not resume vildagliptin. Her pulmonary problems resolved within weeks.

A chest CT performed at the time of admission revealed bilateral, nonsegmental ground-glass opacities in the lower lobes of her lungs (Fig. 1c). Although her respiratory disorder resolved during a course of empirical antibiotic therapy, there was no evidence of bacterial pneumonia. In addition, there was no clinical evidence of any other cause of her respiratory manifestations, such as connective tissue diseases, hypersensitivity pneumonitis secondary to inhaled organic dusts, fungal or viral infection, or hemodynamic disturbances. She had no medication history over the past few months other than 1 day of vildagliptin use. Thus, on the basis of the clinical course and imaging findings, she probably exhibited drug-induced IP [6–8] caused by vildagliptin which she had taken the previous day.

Assessment of cell-mediated immunity using DLST or LMT is a method to confirm the diagnosis of drug-induced disease by detecting drug-sensitized lymphocytes. A study of Japanese patients has shown that the sensitivity of LMT for drug allergies including pulmonary disorders was higher (approximately 60 %) than that of DLST (approximately 20 %), while the specificity of both tests is >90 % [9]. In the present case, our patient showed a negative DLST but a positive LMT for vildagliptin. Although these tests do not have a definite role in the diagnosis of drug-induced lung injury [10], the positive LMT in our patient supported the diagnosis of vildagliptin-induced IP.

The period of drug exposure in previously reported cases of patients with DPP-4 inhibitor-induced lung injury varied from 4 days to 9 months [3–5]. In the present case, our patient developed vildagliptin-induced IP with a drug exposure time of only 1 day. The precise mechanism of her vildagliptin-induced lung injury remains uncertain, but physicians should consider that drug-induced lung injury caused by DPP-4 inhibitor, although rare, can appear acutely even within days after the administration of this drug.

T1D is characterized by the destruction of pancreatic beta cells and insulin-deficient hyperglycemia [11]. The rate of beta-cell destruction is variable from patient to patient and depending on the manner of onset and progression, T1D is classified as fulminant, acute-onset, or slowly progressive in Japan. Our patient developed insulin-deficient hyperglycemia and DKA following 2 months of hyperglycemic symptoms and therefore met the diagnostic criteria for acute-onset T1D [12]. The presence of the class II HLA-DRB1*04:05-DQB1*04:01 haplotype is consistent with her diagnosis of T1D.

She presented with elevated serum pancreatic enzymes without abdominal pain or morphological changes in her pancreas during the weeks after admission. Such elevated pancreatic enzymes are usually observed at the onset of fulminant T1D [13], which is distinguished from acute-onset T1D by the abrupt occurrence of insulin deficiency and DKA within days and the absence of islet-related autoantibodies. She developed acute-onset T1D in the presence of islet-related autoantibodies and never experienced fulminant T1D. Therefore, her elevated serum pancreatic enzymes were probably induced by vildagliptin [1, 6].

Following hyperglycemic symptoms, including thirst, polyuria, and a 10-kg body weight loss over 2 months, she dramatically fell critically ill within 24 hours before admission for acute respiratory failure and severe DKA and fulfilled the criteria for systemic inflammatory response syndrome [14]. DKA is a serious acute metabolic complication of diabetes mellitus characterized by uncontrolled hyperglycemia, metabolic acidosis, and increased total body ketone concentration. Hyperglycemia in patients with DKA is known to be associated with a severe inflammatory state characterized by elevated proinflammatory cytokines [15]. Of importance, respiratory failure aggravates the course of DKA [16]. These findings suggest that in the present case, the development of vildagliptin-induced acute lung injury under conditions of insulin-deficient hyperglycemia likely precipitated and aggravated DKA and resulted in the acute crisis.

Drug-induced IP can occur in patients taking methotrexate, which is an antimetabolite and antifolate drug used to treat cancer and autoimmune disease [17]. Japanese patients with rheumatoid arthritis are known to be more susceptible to methotrexate-induced IP than are other ethnic groups. A genetic study of Japanese patients suggested the presence of HLA-A*31:01 as a predisposing factor for drug-induced IP caused by methotrexate and possibly other drugs [18]. Our patient had never taken methotrexate and did not have rheumatoid arthritis, but there may have been an association between her HLA-A*31:01 and vildagliptin-induced IP.

## Conclusions

We described a Japanese patient with diabetes mellitus who developed drug-induced acute lung injury and elevated serum pancreatic enzymes caused by vildagliptin, both of which were reversible after discontinuation of the drug. The present case suggests that drug-induced lung injury caused by a DPP-4 inhibitor may appear acutely even within days after administration.

## Abbreviations

CRP, C-reactive protein; CT, computed tomography; DKA, diabetic ketoacidosis; DLST, drug-induced lymphocyte stimulation test; DPP-4, dipeptidyl peptidase-4; HbA1c, glycated hemoglobin; HLA, human leukocyte antigen; IP, interstitial pneumonia; KL-6, sialylated carbohydrate antigen Krebs von den Lungen-6; LDH, lactate dehydrogenase; LMT, leukocyte migration test; T1D, type 1 diabetes mellitus; WBC, white blood cell
